# Transcriptomic Changes in Response to Putrescine Production in Metabolically Engineered *Corynebacterium glutamicum*

**DOI:** 10.3389/fmicb.2017.01987

**Published:** 2017-10-17

**Authors:** Zhen Li, Jian-Zhong Liu

**Affiliations:** Institute of Synthetic Biology, Biomedical Center, Guangdong Provincial Key Laboratory of Improved Variety Reproduction in Aquatic Economic Animals and South China Sea Bio-Resource Exploitation and Utilization Collaborative Innovation Center, School of Life Sciences, Sun Yat-sen University, Guangzhou, China

**Keywords:** *Corynebacterium glutamicum*, putrescine, comparative transcriptomic analysis, physiological change, differentially expressed genes

## Abstract

Putrescine is widely used in industrial production of bioplastics, pharmaceuticals, agrochemicals, and surfactants. Although engineered *Corynebacterium glutamicum* has been successfully used to produce high levels of putrescine, the overall cellular physiological and metabolic changes caused by overproduction of putrescine remains unclear. To reveal the transcriptional changes that occur in response to putrescine production in an engineered *C. glutamicum* strain, a comparative transcriptomic analysis was carried out. Overproduction of putrescine resulted in transcriptional downregulation of genes involved in glycolysis; the TCA cycle, pyruvate degradation, biosynthesis of some amino acids, oxidative phosphorylation; vitamin biosynthesis (thiamine and vitamin 6), metabolism of purine, pyrimidine and sulfur, and ATP-, NAD-, and NADPH-consuming enzymes. The transcriptional levels of genes involved in ornithine biosynthesis and NADPH-forming related enzymes were significantly upregulated in the putrescine producing *C. glutamicum* strain PUT-ALE. Comparative transcriptomic analysis provided some genetic modification strategies to further improve putrescine production. Repressing ATP- and NADPH-consuming enzyme coding gene expression via CRISPRi enhanced putrescine production.

## Introduction

Putrescine (1,4-diaminobutane) is widely used as a building block for the industrial production of bioplastics, pharmaceuticals, agrochemicals, and surfactants. For example, putrescine is a raw material used in the production of the bioplastic polyamide nylon-4,6 via polycondensation with adipic acid. Nylon-4,6 is a superior engineering plastic due to its high melting point, high mechanical strength, and excellent solvent resistance. The demand for putrescine is approximately 10,000 tons per year in Europe and is expected to grow (Scott et al., 2007).

The potential commercial demands mean that the efficient biotechnological production of putrescine has become increasingly necessary. After introducing an ornithine decarboxylase gene, putrescine has been produced using engineered *Escherichia coli* (Qian et al., 2009) and *Corynebacterium glutamicum* (Schneider and Wendisch, 2010). An engineered *E. coli* XQ52 (p15SpeC) strain was constructed for putrescine production by a combination of deleting endogenous degradation pathways and replacing the native promoters of the ornithine biosynthetic genes. The strain produced 1.68 g/L of putrescine with a yield of 0.166 g/g glucose in a shake-flask fermentation and 24.2 g/L with a productivity of 0.75 g/L.h in a 6.6-L fed-batch fermentation (Qian et al., 2009). The Wendisch group constructed a series of engineered *C. glutamicum* strains for putrescine production (Schneider and Wendisch, 2010; Schneider et al., 2012; Choi et al., 2014; Nguyen et al., 2015a,b). Their strategies included: (1) lowering the ornithine carbamoyltransferase gene (*argF*) expression by modifications of the *argF* promoter, translational start codon, and ribosome-binding site (Choi et al., 2014); (2) reducing α-ketoglutarate decarboxylase (Kgd) activity by replacing the *kgd* native start codon GTG with TTG and the native *odhI* gene with the *odhI^T15A^* gene; (3) deleting the *snaA* gene to eliminate putrescine acetylation (Nguyen et al., 2015b); (4) overexpression of the putrescine transporter gene (*cgmA*), the glyceraldehyde 3-phosphate dehydrogenase gene (*gap*), the pyruvate carboxylase gene (*pyc*) and the feedback-resistant *N*-acetylglutamate kinase variant gene (*argB*^A49V/M54V^). The final engineered *C. glutamicum* strain NA6 produced 58.1 mM (5.1 g/L) of putrescine with a yield on glucose of 0.26 g/g in a flask culture (Nguyen et al., 2015a), representing the highest values yet seen. The titer and yield of *C. glutamicum* NA6 were 1.99- and 2-fold higher than that of the parent strain *C. glutamicum* PUT21 (Nguyen et al., 2015a), respectively. The parent strain *C. glutamicum* PUT21 produced 19 g/L putrescine with a productivity of 0.55 g/L/h and a yield 0.166 g/g glucose in a fed-batch fermentation (Schneider et al., 2012).

Although engineered *C. glutamicum* has been successfully employed for the high-level production of putrescine, the overall cellular physiological and metabolic changes caused by the overproduction of putrescine remain unclear. Transcriptome analysis has become an effective approach for monitoring cellular physiological and metabolic changes (Yu et al., 2016). Detailed information on cellular physiological changes cannot only allow for a much better understanding of the underlying regulatory mechanisms but also provide new genetic modification strategies for the further improvement in the production of metabolites. Thus, to understand the cellular physiological and metabolic changes occurring in response to the overproduction of putrescine, we carried out a comparative transcriptomic analysis between the putrescine-producer *C. glutamicum* PUT-ALE and the wild-type strain *C. glutamicum* ATCC 13032.

## Materials and Methods

### Strains, Plasmids, and Primers

The bacterial strains used in this study are listed in **Table 1**. Plasmids and primers used in this study are presented in Supplementary Table 1.

**Table 1:** Strains used in this study.

Name	Description	Reference/Sources
Strains		
*Corynebacterium glutamicum* ATCC 13032	Wild-type	ATCC
*C. glutamicum* ΔAPE6937R42	Ornithine producing strain, the evolved strain of *C. glutamicum* ATCC 13032 (Δ*argF*Δ*proB*Δ*speE*), Δ*argR*	Jiang et al., 2013a
*C. glutamicum* PUT-ALE	Putrescine producer, the metabolically evolved strain of *C. glutamicum* ΔAPE6937R42 with Δ*puo*, Δ*fabG*:: P_H36_-*speC1_ECL_*, Δ*butA* and Δ*snaA*	Lab storage
*C. glutamicum* PUT-ALE-KT	Putrescine producer, the *kgd* native GTG start codon in *C. glutamicum* PUT-ALE was replaced with TTG.	This study

### Plasmid Construction

Genes were amplified from genomes using the responding primers (Supplementary Table 1) and cloned into pEC-XK99E (Kirchner and Tauch, 2003). Gene disruption was performed via two-step homologous recombination using the non-replicable integration vector pK-JL as described by Jiang et al. (2013a,b)).

To enhance specificity and reduce off-target effects, the *dcas9* on pCRISPathBrick (Cress et al., 2015) was site-directed mutated into *dcas9 (K848A/K1003A/R1060A)* as described as Slaymaker et al. (2016) to obtain pCRISPathBrick^∗^. The p15A *ori* was amplified from pBAD33 (Guzman et al., 1995) using P15AF and P15AR. The vector backbone was amplified from pEC-XK99E (Kirchner and Tauch, 2003) using primer PEC-AF and PEC-AR. The two PCR products were recombined using ClonExpress II One Step Cloning Kit (Vazyme Biotech Co., Ltd., Nanjing, China) to obtain pEC-XK-p15A. The *dcas9^∗^* gene was amplified from pCRISPathBrick^∗^ using primers dcas9^∗^F and dcas9^∗^R and then cloned into the *XmaI/XbaI* sites of pEC-XK-p15A to generate pEC-dcas9^∗^ (Supplementary Figure 1A). The sgRNA sequence was amplified from pTargetF (Jiang et al., 2015) using primers sgRNAF and sgRNAR. The vector backbone was amplified from pXMJPsod using primers psodGF and psodGR. The two PCR products were recombined using ClonExpress II One Step Cloning Kit to obtain the sgRNA plasmid pXMJPsod-sgRNA.

The pXMJPsod-X-sgRNA series (Supplementary Figure 1B), used in target single-gene repression with a targeting N20 sequence of gene loci of interest, was obtained by inverse PCR using primes the target N20F and PsodG-R from pXMJPsod-sgRNA, and followed by self-ligation.

### Putrescine Production in Shake Flasks

A single colony was inoculated into 5 mL of seed medium in a test tube, which was aerobically cultured overnight at 200 rpm and 30°C. The overnight seed culture was used to inoculate 50 mL of fermentation medium with an initial OD_600_ of 0.2. The primary cultures were incubated at 30°C for 72 h in a rotary shaking incubator at 200 rpm. Each liter of seed medium contained 25 g of glucose, 10 g of yeast extract, 10 g of corn steep liquor, 15 g of (NH_4_)_2_SO_4_, 2.5 g of MgSO_4_ 7H_2_O, 1 g of KH_2_PO_4_, 0.5 g of K_2_HPO_4_, 0.5 g of Na_2_HPO_4_, and 10 g of CaCO_3_. Each liter of fermentation medium contained 100 g of glucose, 20 g of corn steep liquor, 50 g of (NH_4_)_2_SO_4_, 2.5 g of MgSO_4_ 7H_2_O, 1 g of KH_2_PO_4_, 0.5 g of K_2_HPO_4_, 0.5 g of Na_2_HPO_4_, 20 mg of FeSO_4_ 7H_2_O, 20 mg of MnSO_4_ 4H_2_O, 2 g of molasses, 1 mL of Tween-80, and 10 g of CaCO_3_. The initial pH of both media described above was adjusted to 7.0.

### Analysis of Growth and Metabolite Concentration

Growth was monitored by measuring the optical density of the culture at 600 nm after adding 0.2 M HCl to dissolve CaCO_3_. The glucose concentration was determined using glucose oxidase and a glucose assay kit (Shanghai Rongsheng Biotech Corporation, Shanghai, China). The putrescine concentration was determined using a Shimadzu HPLC system (LC-20A HPLC, Shimadzu, Japan) equipped with an Inertsil ODS-SP column (5 μm, 4.6 mm × 150 mm, GL Sciences Inc., Tokyo, Japan) as described by Schneider and Wendisch (2010). Putrescine was first derivatized using 9-fluorenylmethyl chloroformate (FMOC). The fluorescent derivatives were detected by excitation at 263 nm (emission at 310 nm). The mobile phase consisted of solvent A (0.05 M sodium acetate, pH 4.2) and solvent B (acetonitrile) with a flow rate of 1.3 mL/min. The following gradient was used: 0 min, 38% B; 5 min, 38% B; 12 min, 57% B; 14 min, 57% B; 20 min, 65% B; 25 min, 76% B; and 35 min, 76% B. A standard curve was constructed from serial dilutions of a standard stock solution of 1,4-diaminobutane.

### Transcriptome Analysis

RNA-Seq was performed by GENWIZ (Shuzhou, China) using an Illumina HiSeq sequencer (Illumina, San Diego, CA, United States). Each sample was analyzed in duplicate. Cells cultured for 48 h were harvested by centrifugation at 300 rpm for 2 min to remove CaCO_3_ and then at 5,000 × *g* for 15 min and washed twice with PBS. Total RNA was extracted using TRIzol Reagent (Invitrogen) and an RNeasy Mini Kit (Qiagen). Total RNA from each sample was quantified and qualified by an Agilent 2100 Bioanalyzer (Agilent Technologies, Palo Alto, CA, United States), NanoDrop (Thermo Fisher Scientific Inc.) and a 1% agarose gel. One μg of total RNA with RIN values above 7 was used for following library preparation. Next generation sequencing library preparations were performed according to the manufacturer’s protocol (NEBNext^®^ Ultra^TM^ Directional RNA Library Prep Kit for Illumina^®^). The rRNA was depleted from the total RNA using a Ribo-Zero rRNA Removal Kit (Bacteria) (Illumina). The rRNA-depleted mRNA was then fragmented and reverse-transcribed. First-strand cDNA was synthesized using ProtoScript II Reverse Transcriptase with random primers and Actinomycin D. The second-strand cDNA was synthesized using Second Strand Synthesis Enzyme Mix (including dACG-TP/dUTP). The double-stranded cDNA was purified using an AxyPrep Mag PCR Clean-up kit (Axygen) and was then treated with End Prep Enzyme Mix to repair both ends and perform dA-tailing of cDNA in one reaction, followed by a T-A ligation to add adaptors to both ends. Size selection of adaptor-ligated DNA was then performed using an AxyPrep Mag PCR Clean-up kit (Axygen)to recover ∼360 bp fragments (with approximate insert sizes of 300 bp). The dUTP-marked second strand was digested with Uracil-Specific Excision Reagent (USER) enzyme (New England Biolabs). Each sample was then amplified by PCR for 11 cycles using P5 and P7 primers, with both primers carrying sequences that can anneal with the flow cell to perform bridge PCR and the P7 primer carrying a six-base index allowing for multiplexing. The PCR products were purified using an AxyPrep Mag PCR Clean-up kit (Axygen), validated using an Agilent 2100 Bioanalyzer (Agilent Technologies, Palo Alto, CA, United States), and quantified with a Qubit 2.0 Fluorometer (Invitrogen, Carlsbad, CA, United States). Next, libraries with different indices were multiplexed and loaded onto an Illumina HiSeq instrument according to the manufacturer’s instructions (Illumina, San Diego, CA, United States). Sequencing was carried out using a 2x150 paired-end (PE) configuration; image analysis and base calling were conducted using the HiSeq Control Software (HCS) + OLB + GAPipeline-1.6 (Illumina) on the HiSeq instrument. The sequences were processed and analyzed by GENEWIZ.

Raw data were processed to generate the pass filter data by Bcl2fastq (v 2.17.1.14), quality checked using the FastQC (v 0.10.1) tool and finally filtered to prepare the clean reads using Cutadapt (v 1.9.1). The clean data were aligned to the reference genome of *C. glutamicum* ATCC 13032 (Uniprot: UP000000582) using Bowtie 2 (v 2.1.0^1^). The gene transcript expression levels were calculated using HTSeq (v 0.6.1p1) (Anders et al., 2015) and then normalized based on the FPKM (fragments per kilobase of exon per million fragments mapped) method. A false-discovery rate of ≤0.05 (Benjamini and Yekutieli, 2001) and the absolute value of the log_2_ ratio ≥ 1 were applied as threshold values to define a significant difference in gene expression levels using the DESeg2 (v1.6.3) in Bioconductor package (Anders and Huber, 2010). GO-TermFinder (v0.86) (Boyle et al., 2004) was used in identifying Gene Ontology (GO) terms to annotate a list of enriched genes with a significant *p*-value of less than 0.05.

Raw sequence data were deposited in the NCBI Sequence Read Archive database (SRA) under the accession number of SRP117800.

## Results and Discussion

### Culturing of *C. glutamicum* PUT-ALE and Its Parent Strain *C. glutamicum* ΔAPE6937R42

**FIGURE 1:**
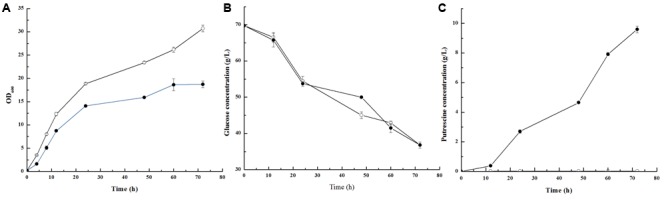
The growth **(A)**, glucose consumption **(B)** and putrescine production **(C)** in *Corynebacterium glutamicum* PUT-ALE (•) and ATCC 13032 (∘). Data represent the average of three replicates and error bars represent standard deviation.

In our previous study, we constructed the *C. glutamicum* PUT-ALE strain from the ornithine producer *C. glutamicum* ΔAPE6937R42 (Jiang et al., 2013a) for putrescine production using a metabolic evolution strategy (Supplementary Text). We first compared the growth, glucose consumption, and putrescine production of the *C. glutamicum* PUT-ALE and the wild-type strain *C. glutamicum* ATCC 13032 strains. As shown in **Figure 1**, *C. glutamicum* PUT-ALE produced a higher level of putrescine than the wild-type strain *C. glutamicum* ATCC 13032. Interestingly, *C. glutamicum* PUT-ALE (μ = 0.38 h^-1^) showed a lower growth rate than *C. glutamicum* ATCC 13032 (μ = 0.43 h^-1^). This may be because *C. glutamicum* PUT-ALE and its parent strain *C. glutamicum* ΔAPE6937R42 are L-arginine and L-proline auxotrophs, resulting from knockouts of the *argF* and *proB* genes (Jiang et al., 2013a,b). The rate of glucose consumption by *C. glutamicum* PUT-ALE was similar to that of the wild-type strain ATCC 13032.

### Transcriptomic Changes

To identify the cellular physiological and metabolic changes occurring in response to the overproduction of putrescine, we analyzed the transcriptomic changes between the putrescine-producer *C. glutamicum* PUT-ALE and the wild-type strain *C. glutamicum* ATCC13032. The putrescine production resulted in the differential expression of 607 genes, of which 283 were upregulated and 324 were downregulated (Supplementary Table 2). We also analyzed the transcriptomic changes between the putrescine-producer *C. glutamicum* PUT-ALE and its parent strain *C. glutamicum* ΔAPE6937R42. A total of 269 genes showed significantly different expression patterns (Supplementary Table 3). Of them, only 29 genes were related to metabolism. Thus, we focused on differentially expressed genes (DEGs) between *C. glutamicum* PUT-ALE and the wild-type strain *C. glutamicum* ATCC13032 in this study.

**FIGURE 2:**
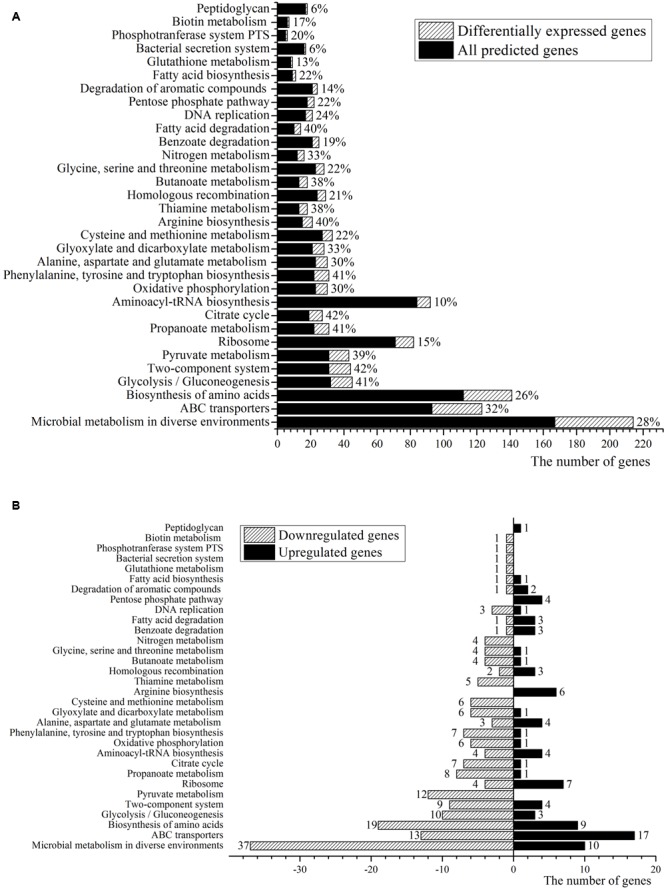
Pathway gene ontology enrichment analysis. **(A)** The ratio of the DEGs in the total number of genes detected. **(B)** The numbers of the DEGs.

The GO project provides a controlled vocabulary to describe gene products within three categories: biological process, molecular function and cellular component (Boyle et al., 2004). GO enrichment analysis has become a commonly used approach for functional studies, and the GO analysis of DEGs can help biologists better understand the functional relevance of DEGs. In **Figure 2**, the results of a GO analysis of DEGs for *C. glutamicum* PUT-ALE vs. ATCC 13032 is presented.

**FIGURE 3:**
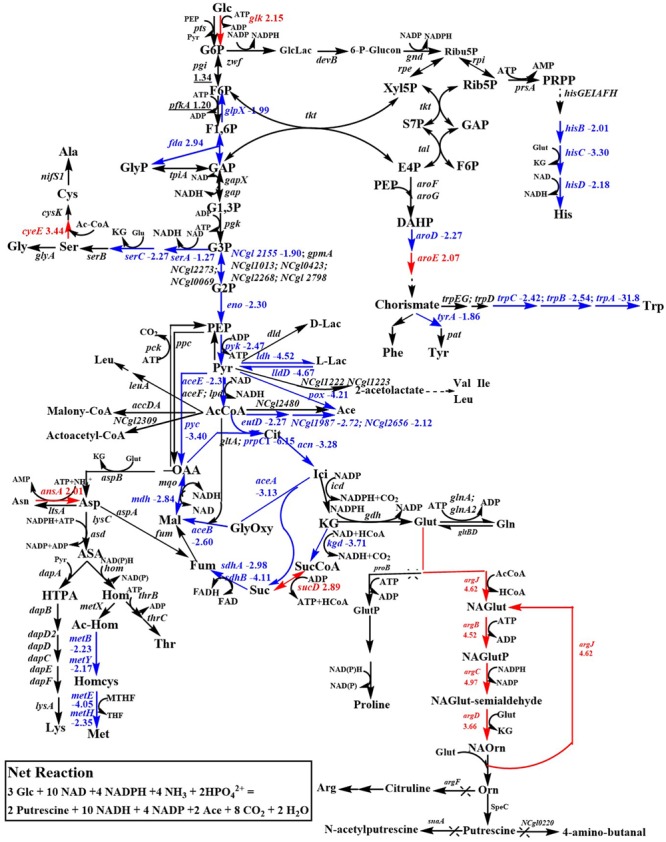
Differentially expressed genes involved in glycolysis, the TCA cycle, pyruvate metabolism, amino acid biosynthesis and the putrescine biosynthetic pathway. The numbers indicate the values of the log_2_ ratios of the expression levels in *C. glutamicum* PUT-ALE vs. *C. glutamicum* ATCC 13032. Red indicates upregulation. Blue indicates downregulation. Glc, glucose; G6P, glucose 6-phosphate; F6P, fructose 6-phosphate; F1,6P, fructose 1,6-bisphosphate; GAP, D-Glyceraldehyde 3-phosphate; GlyP, glycerone phosphate; G1,3P, 1,3-bisphospho-D-glycerate; G3P, 3-phosphoglycerate; G2P, 2-phospho-(R)-glycerate; PEP, phosphoenolpyruvate; Pyr, pyruvate; AcCoA, acetyl-CoA; GlcLac, D-glucono-1,5-lactone 6-phosphate; 6-*P*-glucon, 6-phospho-D-gluconate; Ribu5P, D-Ribulose 5-phosphate; Rib5P, D-ribose 5-phosphate; Xyl5P, D-Xylulose 5-phosphate; S7P, D-sedoheptulose 7-phosphate; E4P, D-erythrose 4-phosphate; PRPP, 5-phosphoribosyl diphosphate; His, L-histidine; DAHP, 3-deoxy-arabino-heptulonate 7-phosphate; Trp, L-tryptophan; Phe, L-phenylalanine; Tyr: L-tyrosine; D-Lac, D-Lactate; L-Lac, L-lactate; Ace, acetate; Val, L-valine; Ile, L-isoleucine; Leu, L-leucine; Ser, L-serine; Gly, L-glycine; Cys, L-cysteine; Ala, L-alanine; Cit, citrate; Ici, isocitrate; KG, 2-oxoglutarate; SucCoA, succinyl-CoA; Suc, succinate; Fum, fumarate; Mal, malate; OAA, oxaloacetate; Asp, L-aspartate; Asn, L-asparagine; ASA, L-aspartate 4-semialdehyde; HTPA, (2S,4S)-4-hydroxy-2,3,4,5-tetrahydrodipicolinate; Lys, L-lysine; Hom, homoserine; Thr, L-threonine; Ac-Hom, *O*-acetylhomoserine; Homcys, L-homocysteine; Met, L-methionine. Glut, L-glutamate; Gln, L-glutamine; GlutP, L-glutamate 5-phosphate; NAGlut, *N*-acetylglutamate; NAGlutP, *N*-acetyl-glutamyl 5-phosphate; NAGlut-semialdehyde, *N*-acetylglutamate semialdehyde; NAOrn, *N*-acetyl-ornithine; Orn, ornithine; Arg, L-arginine.

**FIGURE 4:**
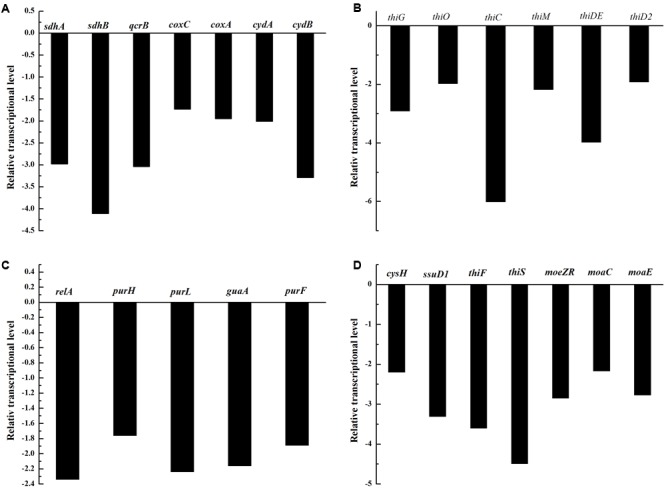
The relative transcriptional levels of genes involved in oxidative phosphorylation **(A)**, vitamin biosynthesis **(B)**, the metabolism of purine and pyrimidine **(C)**, and sulfur metabolism **(D)**.

DEGs involved in metabolic pathways are presented in **Figures 3** and **4**. As shown in **Figure 3**, most of the genes (*glpX, fda, gpmB, eno, pyk, aceE, prpC1, acn, kgd, sdhAB, mdh, aceAB*) involved in the glycolysis and tricarboxylic acid (TCA) cycle were significantly downregulated in *C. glutamicum* PUT-ALE compared to *C. glutamicum* ATCC13032. The low rate of growth of *C. glutamicum* PUT-ALE is consistent with the observed downregulated data. The *pyc* gene in *C. glutamicum* PUT-ALE was also downregulated. The pyruvate carboxylase encoded by *pyc* is one of the most important anaplerotic enzymes in *C. glutamicum*. Overexpression of the *pyc* gene can drive greater EMP flux into the TCA cycle to strengthen it. It has been demonstrated that overexpression of the *pyc* gene increased L-glutamate (Shirai et al., 2007; Hasegawa et al., 2008), L-arginine (Man et al., 2016b) and putrescine (Nguyen et al., 2015a) production in *C. glutamicum*. Thus, we expressed *pyc* or its mutant *pyc458* from a plasmid in *C. glutamicum* PUT-ALE. As shown in **Table 2**, overexpression of the native *pyc* gene slightly increased putrescine production, while overexpression of the mutated *pyc458* gene markedly increased putrescine production by 16% to 133.51 ± 7.20 mM. It has been reported that *pyc458* is a beneficial mutation for L-lysine production (Ohnishi et al., 2002).

**Table 2:** Effect of the *pyc* and *kgd* gene on putrescine production in *C. glutamicum* PUT-ALE.

Strain	OD_600_	Putrescine (mM)	Yield (%, g/g)
*C. glutamicum* PUT-ALE (pEC-XK99E)	19.41 ± 0.75	115.12 ± 2.42	27.0 ± 0.1
*C. glutamicum* PUT-ALE (pEC-pyc)	15.93 ± 0.35	123.18 ± 2.71	27.3 ± 0.6
*C. glutamicum* PUT-ALE (pEC-pyc458)	16.98 ± 0.44	133.51 ± 7.20	28.1 ± 1.5
*C. glutamicum* PUT-ALE	17.64 ± 0.27	107.95 ± 2.31	27.6 ± 1.1
*C. glutamicum* PUT-ALE-KT	20.04 ± 0.78	114.39 ± 2.14	28.4 ± 1.5

*Data represent the average of three replicates and error bars represent standard deviation.*

The transcription level of the *kgd* gene was also downregulated in *C. glutamicum* PUT-ALE. Alpha-ketoglutarate (KG) is a key node of the TCA cycle, and α-ketoglutarate decarboxylase (encoded by *kgd*) catalyzes the oxidative decarboxylation of KG to synthesize succinyl coenzyme A. The downregulation of *kgd* transcription can channel increased carbon flux into the glutamate biosynthetic pathway, enhancing putrescine production. Many groups have reported that decreasing the Kgd activity in *Corynebacterium*, or even deleting *kgd*, increased the production of glutamate (Asakura et al., 2007; Kim et al., 2009), the glutamate-derived compound putrescine (Nguyen et al., 2015a), gamma-aminobutyric acid (Jorge et al., 2017) and L-arginine (Chen et al., 2015; Man et al., 2016b). It has been demonstrated that the exchanging the translational start codon of the *kgd* gene from GTG to TTG reduced the Kgd activity from 11 to 7 mU/mg (Nguyen et al., 2015a). Thus, we replaced the native GTG start codon of the *C. glutamicum* PUT-ALE *kgd* gene with TTG to obtain *C. glutamicum* PUT-ALE-KT. The resulting strain (*C. glutamicum* PUT-ALE-KT) produced a higher level of putrescine (114.39 ± 2.14 mM) than *C. glutamicum* PUT-ALE (107.95 ± 2.31, **Table 2**), indicating that decreasing the activity of Kgd may be a strategy for further improving putrescine production.

In **Figure 3**, it is observed that may genes that are involved in pyruvate metabolism were significantly down-regulated in *C. glutamicum* PUT-ALE, such as *ldh, lldD, pox, eutD, acyP*, and *ackA*. The downregulation of pyruvate metabolism can drive carbon flux toward glycolysis for putrescine biosynthesis. Genes involved in the putrescine biosynthetic pathway, such as *argJ, argB, argC*, and *argD* were significantly upregulated in *C. glutamicum* PUT-ALE (**Figure 3**).

We also observed that some genes involved in the serine, methionine, histidine, tryptophan, and tyrosine biosynthetic pathway were significantly downregulated (**Figure 3**). These genes include *serA, serC, metB, metY, metE, metH, hisB, hisC, hisD, aroD, trpC, trpB, trpA*, and *tyrA.* The enzyme encoded by *serC* or *hisC* catalyzes the glutamate-consuming reaction. The downregulation of *serC* and *hisC* transcription may provide more glutamate for putrescine biosynthesis.

As shown in **Figure 4A**, the transcriptional levels of genes involved in oxidative phosphorylation were down-regulated, such as *sdhA, sdhB, qcrB, coxC, coxA, cydA*, and *cydB*. Genes involved in thiamine and vitamin B6 biosynthesis, such as *thiG, thiO, thiC, thiM, thiDE*, and *thiD2*, were also down-regulated (**Figure 4B**). The transcriptional levels of genes involved in purine and pyrimidine metabolism, such as *relA, purH, purL, guaA*, and *purF* were down-regulated (**Figure 4C**), as were genes involved in sulfur metabolism, such as *cysH, ssuD1, thiF, thiS, moeZR, moaC*, and *moaE* (**Figure 4D**). Of the above genes, *thiM, thiDE, thiD2, relA, purl, guaA*, and *moeZR* encode adenosine triphosphate (ATP)-consuming enzymes. The transcriptional downregulation of these genes could result in more ATP being available for putrescine production.

ATP is the most important energy source for metabolic reaction and pathways, playing an important role in cell growth and the production of target metabolites. Many ATP-consuming enzyme encoding genes, such as *rbsK, cysD, cysN, pknG, pknB, bioD, iolC, mthfs, coaE, chlI, glgC*, and *moeZR*, were downregulated in *C. glutamicum* PUT-ALE (Supplementary Table 2). It has been reported that increasing the ATP supply enhanced L-arginine production in *C. glutamicum* (Man et al., 2016a). The protein kinases encoded by *pknG* and *pknB* phosphorylate the α-ketoglutarate decarboxylase inhibitor OdhI, and unphosphorylated OdhI inhibits α-ketoglutarate decarboxylase activity (Niebisch et al., 2006; Schultz et al., 2009; Raasch et al., 2014). Thus, the decreased transcription of *pknG* and *pknB* in *C. glutamicum* PUT-ALE may increase the ability of OdhI to inhibit α-ketoglutarate decarboxylase. The regulation of OdhI phosphorylation by the deletion of the protein kinase encoding gene *pknG* has been previously shown to increase glutamate production (Schultz et al., 2007).

In **Figure 3**, it is observed that synthesizing one mole of putrescine requires 2 moles of NADPH and 5 moles of NAD. Thus, NADPH availability and transhydrogenation between NAD and NADP are important for putrescine production. The transcriptional levels of the NADPH-consuming enzyme encoding genes [*rhcM2* and NAD (FAD)-dependent dehydrogenase gene *NCgl2615*] and the NAD-consuming enzyme encoding genes (*gabD3, iolG*, and *fdhF*) were significantly downregulated. The transcriptional levels of NADPH-forming enzyme encoding genes, such as *proA, aldH*, and *mdhB*, were significantly upregulated in *C. glutamicum* PUT-ALE (Supplementary Table 2). The expression patterns can increase NADPH or NAD availability for putrescine production. It has been demonstrated that increasing NADPH availability enhances L-ornithine production (Jiang et al., 2013b; Hwang and Cho, 2014; Kim et al., 2015).

CRISPRi system is a powerful tool to repress expression of targeted genes (Qi et al., 2013). It has successfully applied to repress genes for improving L-lysine and L-glutamate production in *C. glutamicum* (Cleto et al., 2016). Thus, we established a CRISPRi system, which contains the *dcas9 (K848A/K1003A/R1060A)* plasmid pEC-dcas9^∗^ (Supplementary Figure 1A) and the sgRNA plasmid pXMJPsod-X-sgRNA (Supplementary Figure 1B). To validate the effects of ATP- and NADPH-consuming enzyme genes, we applied the CRISPRi system to repress expression of ATP- and NADPH-consuming enzyme encoding genes in *C. glutamicum* PUT-ALE. The results were presented in **Table 3**. Repressing ATP-consuming enzyme encoding genes, such as *carB, xylB, accDA, purL, coaA, pknG*, and *panC2* resulted in increasing putrescine production of 5–10%. Repressing the *dxr, aroE*, or *trxB* expression enhanced putrescine production by 13, 19, or 20%, respectively. The *dxr* encodes 1-deoxy-D-xylulose 5-phosphate reductoisomerase which catalyzes the reduction of 1-deoxy-D-xylulose 5-phosphate to 2-C-methyl-D-erythritol 4-phosphate in the presence of NADPH. The *aroE* encodes shikimate dehydrogenase which catalyzes NAD^+^-dependent oxidation of shikimate to 3-dehydroshikimate. The *trxB* encodes thioredoxin reductase which catalyzes the reduction of thioredoxin disulfide to thioredoxin in the presence of NADPH. Repressing the *dxr, trxB*, or *aroE* expression can provide more NADPH or NAD for putrescine production.

**Table 3:** Effect of perturbations of ATP- and NADPH-consuming enzyme encoding genes on putrescine production in *C. glutamicum* PUT-ALE.

Gene targeted	Gene encoding enzyme/protein	OD_600_	Putrescine (mM)	Ratio
**ATP-consuming**				
Control		16.83 ± 0.18	103.66 ± 3.29	1.00
*carB*	Carbamoyl-phosphate synthase large subunit [EC6.3.5.5]	17.27 ± 0.20	109.00 ± 4.36	1.05
*thrB1*	Homoserine kinase [EC 2.7.1.39]	17.02 ± 0.20	105.05 ± 0.45	1.01
*coaA*	Pantothenate kinase [EC 2.7.1.33],	16.56 ± 0.51	110.18 ± 1.32	1.06
*glnA*	Glutamine synthetase [EC 6.3.1.2]	15.63 ± 0.06	86.44 ± 5.17	0.86
*nadD*	Nicotinate-nucleotide adenylyltransferase [EC 2.7.7.18]	16.71 ± 0.18	95.68 ± 0.93	0.92
*hemH*	Phosphoribosylaminoimidazole-succinocarboxamide synthase [EC 6.3.2.6],	16.55 ± 0.35	86.74 ± 8.33	0.84
*xylB*	Xylulokinase [EC 2.7.1.17]	16.62 ± 0.42	108.88 ± 0.07	1.05
*guaA*	GMP synthase (glutamine-hydrolysing) [EC:6.3.5.2],	17.25 ± 0.24	82.71 ± 1.40	0.80
*accBC*	Acyl-CoA carboxylase	18.26 ± 0.42	80.20 ± 0.55	0.77
*accDA*	Acetyl-CoA carboxylase beta subunit [EC 6.4.1.2]	17.36 ± 0.10	109.24 ± 0.04	1.05
*purL*	Phosphoribosylformylglycinamidine synthase [EC 6.3.5.3]	16.60 ± 0.54	108.86 ± 0.59	1.05
*purQ*	Phosphoribosylformylglycinamidine synthase [EC 6.3.5.3]	17.91 ± 0.38	103.78 ± 5.31	1.00
*panC1*	Pantoate-beta-alanine ligase [EC 6.3.2.1]	18.98 ± 1.34	107.24 ± 0.98	1.03
*panC2*	Pantoate-beta-alanine ligase [EC 6.3.2.1]	17.98 ± 0.58	113.94 ± 2.98	1.10
*pknG*	Serine/threonine protein kinases [EC 2.7.11.1]	17.59 ± 0.35	109.57 ± 1.21	1.06
**NADPH-consuming**		
Control		19.80 ± 0.30	108.99 ± 2.51	1.00
*pobA*	*p*-hydroxybenzoate 3-monooxygenase [EC 1.14.13.2]	15.90 ± 0.24	105.36 ± 2.53	0.93
*aldH*	2,5-dioxopentanoate dehydrogenase [EC 1.2.1.26]	16.07 ± 0.15	92.65 ± 3.83	0.88
*fabG1*	3-oxoacyl-[acyl-carrier protein] reductase [EC 1.1.1.100],	16.08 ± 0.26	108.96 ± 0.79	0.96
*adhC*	Maleylacetate reductase [EC 1.3.1.32]	15.62 ± 0.16	101.45 ± 2.38	0.93
*gor*	Dihydrolipoamide dehydrogenase/glutathione oxidoreductase and related enzymes, [EC 1.8.1.7]	15.52 ± 0.35	94.92 ± 3.05	0.87
*dxr*	1-deoxy-D-xylulose-5-phosphate reductoisomerase [EC 1.1.1.267]	15.36 ± 0.50	123.18 ± 0.55	1.13
*asd*	aspartate-semialdehyde dehydrogenase [EC 1.2.1.11]	15.54 ± 0.18	99.7 ± 1.48	0.91
*proA*	Glutamate-5-semialdehyde dehydrogenase [EC 1.2.1.41]	16.21 ± 0.19	98.51 ± 0.87	0.90
*NCgl2558*	Transcriptional regulators	15.87 ± 0.12	99.06 ± 1.92	0.87
*thyX*	Thymidylate synthase (FAD) [EC 2.1.1.148]	19.77 ± 0.48	103.71 ± 3.55	0.95
*aroE*	Shikimate dehydrogenase [EC 1.1.1.25],	16.74 ± 0.06	129.29 ± 1.76	1.19
*sir*	Sulfite reductase (ferredoxin) [EC 1.8.7.1]	17.82 ± 0.30	106.87 ± 2.02	0.98
*NCgl0503*	Aldo/keto reductases	15.18 ± 2.58	110.53 ± 3.30	1.01
*ddh*	diaminopimelate dehydrogenase [EC 1.4.1.16]	15.93 ± 0.09	98.82 ± 2.68	0.91
*ilvC*	Ketol-acid reductoisomerase [EC 1.1.1.86]	16.32 ± 0.30	102.49 ± 3.71	0.94
*qor*	NADPH:quinone reductase and related Zn-dependent oxidoreductases [EC 1.6.5.5]	16.83 ± 0.27	108.78 ± 0.34	0.98
*trxB*	Thioredoxin reductase (NADPH) [EC 1.8.1.9]	17.16 ± 0.36	131.12 ± 0.88	1.20
*NCgl0200*	NADPH:quinone reductase and related Zn-dependent oxidoreductases	16.26 ± 0.24	109.30 ± 0.76	0.99

*Data represent the average of three replicates and error bars represent standard deviation.*

A total of 76 secretion and membrane transport protein encoding genes were significantly differentially expressed in *C. glutamicum* PUT-ALE (Supplementary Table 2). Of these genes, 30 were downregulated and 46 were upregulated. The differential expression may affect the metabolite transport. It has been previously shown that CgmA is a putrescine export permease and that overexpression of the *cgmA* gene increased putrescine production in *C. glutamicum* (Nguyen et al., 2015a,b). We also observed that the transcriptional of the *cgmA* gene in *C. glutamicum* PUT-ALE was significantly upregulated (Supplementary Table 2). A total of 30 transcription factors were significantly differentially expressed in *C. glutamicum* PUT-ALE (Supplementary Table 2). Of these genes, 13 were downregulated and 17 were upregulated. In addition, 378 other genes, such as unknown, transposase and ribosomal RNA genes, were significantly differentially expressed in *C. glutamicum* PUT-ALE (Supplementary Table 2). Of these genes, 189 were downregulated and 189 were upregulated.

## Conclusion

We comparatively analyzed the transcriptomic changes in response to putrescine production in the strain *C. glutamicum* PUT-ALE. The overproduction of putrescine resulted in the transcriptional downregulation of genes involved in: glycolysis, the TCA cycle, pyruvate degradation, the biosynthesis of some amino acids, oxidative phosphorylation, vitamin biosynthesis (thiamine and vitamin 6), the metabolism of purine, pyrimidine and sulfur; and ATP-, NAD- and NADPH-consuming enzymes. The transcriptional levels of genes involved in ornithine biosynthesis and those encoding NADPH-forming enzymes were upregulated in the putrescine producer *C. glutamicum* PUT-ALE. The comparative transcriptomic analysis provided some genetic modification strategies for further improving putrescine production. Overexpression of *pyc* or its mutant *pyc458*, and replacing the *kgd* native start codon GTG with TTG further improved putrescine production. Repressing ATP- and NADPH-consuming enzyme coding gene expression via CRISPRi also enhanced putrescine production. To the best of our knowledge, this is the first report on increasing putrescine production via repressing ATP- and NADPH-consuming enzyme coding gene expression.

## Author Contributions

ZL performed the experiments. J-ZL directed the project and wrote the paper.

## Conflict of Interest Statement

The authors declare that the research was conducted in the absence of any commercial or financial relationships that could be construed as a potential conflict of interest.

## Footnotes

1 http://bowtie-bio.sourceforge.net/bowtie2/manual.shtml

## References

[B1] Anders S. Huber W. (2010). Differential expression analysis for sequence count data. *Genome Biol.* 11:R106. 10.1186/gb-2010-11-10-r106 20979621 PMC3218662

[B2] Anders S. Pyl P. T. Huber W. (2015). HTSeq-a Python framework to work with high-throughput sequencing data. *Bioinformatics* 31 166–169. 10.1093/bioinformatics/btu638 25260700 PMC4287950

[B3] Asakura Y. Kimura E. Usuda Y. Kawahara Y. Matsui K. Osumi T. (2007). Altered metabolic flux due to deletion of *odhA* causes L-glutamate overproduction in *Corynebacterium glutamicum*. *Appl. Environ. Microbiol.* 73 1308–1319. 10.1128/AEM.01867-06 17158630 PMC1828640

[B4] Benjamini Y. Yekutieli D. (2001). The control of the false discovery rate in multiple testing under dependency. *Ann. Stat.* 29 1165–1188. 10.1186/1471-2105-9-114 18298808 PMC2375137

[B5] Boyle E. I. Weng S. A. Gollub J. Jin H. Botstein D. Cherry J. M. (2004). GO::termfinder – open source software for accessing gene ontology information and finding significantly enriched gene ontology terms associated with a list of genes. *Bioinformatics* 20 3710–3715. 10.1093/bioinformatics/bth456 15297299 PMC3037731

[B6] Chen M. L. Chen X. L. Wan F. Zhang B. Chen J. C. Xiong Y. H. (2015). Effect of Tween 40 and DtsR1 on L-arginine overproduction in *Corynebacterium crenatum*. *Microb. Cell Fact.* 14 119. 10.1186/s12934-015-0310-9 26264811 PMC4534012

[B7] Choi H. Kyeong H. H. Choi J. M. Kim H. S. (2014). Rational design of ornithine decarboxylase with high catalytic activity for the production of putrescine. *Appl. Microbiol. Biotechnol.* 98 7483–7490. 10.1007/s00253-014-5669-8 24706212

[B8] Cleto S. Jensen J. V. K. Wendisch V. F. Lu T. K. (2016). *Corynebacterium glutamicum* metabolic engineering with CRISPR interference (CRISPRi). *ACS Synth. Biol.* 5 375–385. 10.1021/acssynbio.5b00216 26829286 PMC4877668

[B9] Cress B. F. Toparlak O. D. Guleria S. Lebovich M. Stieglitz J. T. Englaender J. A. (2015). CRISPathBrick: modular combinatorial assembly of type II-A CRISPR arrays for dCas9-mediated multiplex transcriptional repression in *E. coli*. *ACS Synth. Biol.* 4 987–1000. 10.1021/acssynbio.5b00012 25822415

[B10] Guzman L. M. Belin D. Carson M. J. Beckwith J. (1995). Tight regulation, modulation, and high-level expression by vectors containing the arabinose PBAD promoter. *J. Bacteriol.* 177 4121–4130. 10.1128/jb.177.14.4121-4130.1995 7608087 PMC177145

[B11] Hasegawa T. Hashimoto K. I. Kawasaki H. Nakamatsu T. (2008). Changes in enzyme activities at the pyruvate node in glutamate-overproducing *Corynebacterium glutamicum*. *J. Biosci. Bioeng.* 105 12–19. 10.1263/jbb.105.12 18295714

[B12] Hwang G. H. Cho J. Y. (2014). Enhancement of L-ornithine production by disruption of three genes encoding putative oxidoreductases in *Corynebacterium glutamicum*. *J. Ind. Microbiol. Biotechnol.* 41 573–578. 10.1007/s10295-013-1398-8 24402505

[B13] Jiang L. Y. Chen S. G. Zhang Y. Y. Liu J. Z. (2013a). Metabolic evolution of *Corynebacterium glutamicum* for increased production of L-ornithine. *BMC Biotechnol.* 13:47. 10.1186/1472-6750-13-47 23725060 PMC3681597

[B14] Jiang L. Y. Zhang Y. Y. Li Z. Liu J. Z. (2013b). Metabolic engineering of *Corynebacterium glutamicum* for increasing the production of L-ornithine by increasing NADPH availability. *J. Ind. Microbiol. Biotechnol.* 40 1143–1151. 10.1007/s10295-013-1306-2 23836141

[B15] Jiang Y. Chen B. Duan C. Sun B. Yang J. Yang S. (2015). Multigene editing in the *Escherichia coli* genome via the CRISPR-Cas9 system. *Appl. Environ. Microbiol.* 81 2506–2514. 10.1128/AEM.04023-14 25636838 PMC4357945

[B16] Jorge J. M. P. Nguyen A. Q. D. Perez-Garcia F. Kind S. Wendisch V. F. (2017). Improved fermentative production of gamma-aminobutyric acid via the putrescine route: systems metabolic engineering for production from glucose, amino sugars, and xylose. *Biotechnol. Bioeng.* 114 862–873. 10.1002/bit.26211 27800627

[B17] Kim J. Hirasawa T. Sato Y. Nagahisa K. Furusawa C. Shimizu H. (2009). Effect of odhA overexpression and odhA antisense RNA expression on Tween-40-triggered glutamate production by *Corynebacterium glutamicum*. *Appl. Microbiol. Biotechnol.* 81 1097–1106. 10.1007/s00253-008-1743-4 18923827

[B18] Kim S. Y. Lee J. Lee S. Y. (2015). Metabolic engineering of *Corynebacterium glutamicum* for the production of L-ornithine. *Biotechnol. Bioeng.* 112 416–421. 10.1002/bit.25440 25163446

[B19] Kirchner O. Tauch A. (2003). Tools for genetic engineering in the amino acid-producing bacterium *Corynebacterium glutamicum*. *J. Biotechnol.* 104 287–299. 10.1016/S0168-1656(03)00148-2 12948646

[B20] Man Z. W. Rao Z. M. Xu M. J. Guo J. Yang T. W. Zhang X. (2016a). Improvement of the intracellular environment for enhancing L-arginine production of *Corynebacterium glutamicum* by inactivation of H_2_O_2_-forming flavin reductases and optimization of ATP supply. *Metab. Eng.* 38 310–321. 10.1016/j.ymben.2016.07.009 27474351

[B21] Man Z. W. Xu M. J. Rao Z. M. Guo J. Yang T. W. Zhang X. (2016b). Systems pathway engineering of *Corynebacterium crenatum* for improved L-arginine production. *Sci. Rep.* 6:28629. 10.1038/srep28629 27338253 PMC4919616

[B22] Nguyen A. Q. D. Schneider J. Reddy G. K. Wendisch V. F. (2015a). Fermentative production of the diamine putrescine: system metabolic engineering of *Corynebacterium glutamicum*. *Metabolites* 5 211–231. 10.3390/metabo5020211 25919117 PMC4495370

[B23] Nguyen A. Q. D. Schneider J. Wendisch V. F. (2015b). Elimination of polyamine N-acetylation and regulatory engineering improved putrescine production by *Corynebacterium glutamicum*. *J. Biotechnol.* 201 75–85. 10.1016/j.jbiotec.2014.10.035 25449016

[B24] Niebisch A. Kabus A. Schultz C. Weil B. Bott M. (2006). Corynebacterial protein kinase G controls 2-oxoglutarate dehydrogenase activity via the phosphorylation status of the OdhI protein. *J. Biol. Chem.* 281 12300–12307. 10.1074/jbc.M512515200 16522631

[B25] Ohnishi J. Mitsuhashi S. Hayashi M. Ando S. Yokoi H. Ochiai K. (2002). A novel methodology employing *Corynebacterium glutamicum* genome information to generate a new L-lysine-producing mutant. *Appl. Microbiol. Biotechnol.* 58 217–223. 10.1007/s00253-001-0883-6 11876415

[B26] Qi L. S. Larson M. H. Gilbert L. A. Doudna J. A. Weissman J. S. Arkin A. P. (2013). Repurposing CRISPR as an RNA-guided platform for sequence-specific control of gene expression. *Cell* 152 1173–1183. 10.1016/j.cell.2013.02.022 23452860 PMC3664290

[B27] Qian Z. G. Xia X. X. Lee S. Y. (2009). Metabolic engineering of *Escherichia coli* for the production of putrescine: a four carbon diamine. *Biotechnol. Bioeng.* 104 651–662. 10.1002/bit.22502 19714672

[B28] Raasch K. Bocola M. Labahn J. Leitner A. Eggeling L. Bott M. (2014). Interaction of 2-oxoglutarate dehydrogenase OdhA with its inhibitor OdhI in *Corynebacterium glutamicum*: mutants and a model. *J. Biotechnol.* 191 99–105. 10.1016/j.jbiotec.2014.05.023 24905147

[B29] Schneider J. Eberhardt D. Wendisch V. F. (2012). Improving putrescine production by *Corynebacterium glutamicum* by fine-tuning ornithine transcarbamoylase activity using a plasmid addiction system. *Appl. Microbiol. Biotechnol.* 95 169–178. 10.1007/s00253-012-3956-9 22370950

[B30] Schneider J. Wendisch V. F. (2010). Putrescine production by engineered *Corynebacterium glutamicum*. *Appl. Microbiol. Biotechnol.* 88 859–868. 10.1007/s00253-010-2778-x 20661733

[B31] Schultz C. Niebisch A. Gebel L. Bott M. (2007). Glutamate production by *Corynebacterium glutamicum*: dependence on the oxoglutarate dehydrogenase inhibitor protein OdhI and protein kinase PknG. *Appl. Microbiol. Biotechnol.* 76 691–700. 10.1007/s00253-007-0933-9 17437098

[B32] Schultz C. Niebisch A. Schwaiger A. Viets U. Metzger S. Bramkamp M. (2009). Genetic and biochemical analysis of the serine/threonine protein kinases PknA, PknB, PknG and PknL of *Corynebacterium glutamicum*: evidence for non-essentiality and for phosphorylation of OdhI and FtsZ by multiple kinases. *Mol. Microbiol.* 74 724–741. 10.1111/j.1365-2958.2009.06897.x 19788543 PMC2784874

[B33] Scott E. Peter F. Sanders J. (2007). Biomass in the manufacture of industrial products – the use of proteins and amino acids. *Appl. Microbiol. Biotechnol.* 75 751–762. 10.1007/s00253-007-0932-x 17387469 PMC1914281

[B34] Shirai T. Fujimura K. Furusawa C. Nagahisa K. Shioya S. Shimizu H. (2007). Study on roles of anaplerotic pathways in glutamate overproduction of *Corynebacterium glutamicum* by metabolic flux analysis. *Microb. Cell Fact.* 6 19. 10.1186/1475-2859-6-19 17587457 PMC1919393

[B35] Slaymaker I. M. Gao L. Y. Zetsche B. Scott D. A. Yan W. X. Zhang F. (2016). Rationally engineered Cas9 nucleases with improved specificity. *Science* 351 84–88. 10.1126/science.aad5227 26628643 PMC4714946

[B36] Yu X. L. Jin H. Y. Cheng X. L. Wang Q. Qi Q. S. (2016). Transcriptomic analysis for elucidating the physiological effects of 5-aminolevulinic acid accumulation on *Corynebacterium glutamicum*. *Microbiol. Res.* 192 292–299. 10.1016/j.micres.2016.08.004 27664748

