# Supplementary material for: Transcriptomic Changes in Response to Putrescine Production in Metabolically Engineered Corynebacterium glutamicum
Source: Front Microbiol. 2017 Oct 17;8:1987. doi: 10.3389/fmicb.2017.01987 (PMC5650995; doi:10.3389/fmicb.2017.01987)
Supplement: Supplementary file 1 [file Table_1.DOCX]

**Transcriptomic changes in response to putrescine production in** **metabolically engineered *Corynebacterium glutamicum***

*Zhen Li, Jian-Zhong Liu**

*Biotechnology Research Center and Biomedical Center,* *Guangdong Province Key Laboratory for Aquatic Economic Animals and South China Sea Bio-Resource Exploitation and Utilization Collaborative Innovation Center,* *School of Life Sciences, Sun Yat-sen University, Guangzhou 510275, China*

*Corresponding author: Biotechnology Research Centre, School of Life Science, Sun Yat-Sen University, Guangzhou 510275, P.R. China. Phone: +86-20-84110115. Fax: +86-20-84036461. *Email address*: lssljz@mail.sysu.edu.cn (J. Z. Liu)

**Construction *C. glutamicum* PUT-ALE**

The putrescine oxidase gene was first deleted in the ornithine producer *C. glutamicum* ΔAPE6937R42 (Jiang et al., 2013a) to obtain *C. glutamicum* Δ5. And then the *fabG* gene of *C. glutamicum* Δ5 was replaced with the decarboxylase gene *speC1* from *Enterobacter cloacae* under the control of the strong promoter H36 (Yim et al., 2013) to obtain putrescine producer *C. glutamicum* PUT. Subsequently, the *butA* and *snaA* genes in *C. glutamicum* PUT were deleted to obtain *C. glutamicum* PUT4. Finally, *C. glutamicum* PUT4 was subjected to adaptive evolution driven by growth-based selection to improve putrescine production. After 24 days of the adaptive evolution, one of clones, referred to as *C. glutamicum* PUT-ALE was selected, which produced an 80% higher amount of putrescine compared to *C. glutamicum* PUT4.


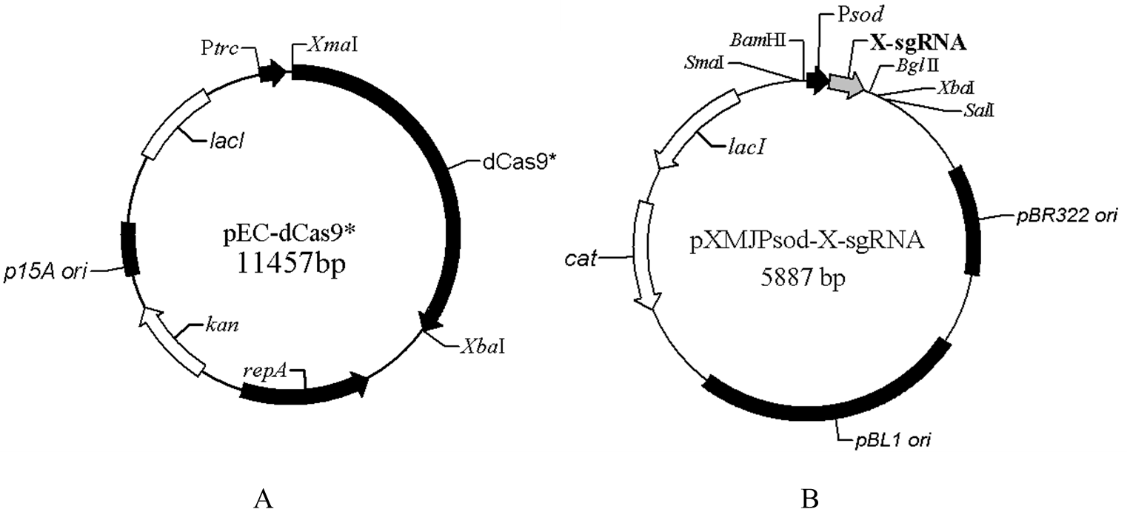


Suppl Fig.1 Plasmids in CRISPRi system. A, the *dcas9* *(K848A/K1003A/R1060A)* plasmid; B, the sgRNA plasmid

Suppl. Table 1 Plasmids and primers used in this study

| Plasmids | | | | | | |
| --- | --- | --- | --- | --- | --- | --- |
| pEC-XK99E | | *C*. *glutamicum*-*E*. *coli* shuttle expression vector, Kan^r^ | Kirchner and Tauch, 2003 | | | |
| pK-JL | | pK18mobsacB derivative, *sacB* is under the control of the *tac-M* promoter, Km^r^, | Jiang et al., 2013a, b | | | |
| pEC-pyc | | pEC-XK99E containing the *C. glutamicum pyc* gene | This study | | | |
| pEC-pyc458 | | pEC-XK99E containing the *C. glutamicum pyc^Pro458Ser^* gene | This study | | | |
| pEC-cgmA | | pEC-XK99E containing the *C. glutamicum cgmA* gene | This study | | | |
| pCRISPathBrick | | Expressing dcas9, p15A ori, Cm^r^, Addgene #65006 | Cress et al., 2015 | | | |
| pBAD33 | | Expression vector, pBAD promoter, p15A *ori*, Cm^r^ | Guzman et al., 1995 | | | |
| pXMJPsod | | *C*. *glutamicum*-*E*. *coli* shuttle expression vector pXMJ19 derivative. The *tac* promoter and lacI^q^ of pXMJ19 was replaced with *C. glutamicum sod* promoter | Lab storage | | | |
| pTarget | | *E. coli* sgRNA expression vector | Jiang et al., 2015 | | | |
| pCRISPathBrick* | | Expressing dcas9(K848A/K1003A/R1060A), p15A ori, Cm^r^, | This study | | | |
| pEC-XK-p15A | | *C*. *glutamicum*-*E*. *coli* shuttle expression vector, pEC-XK99E derivative, p15A ori | This study | | | |
| pEC-dcas9* | | *C*. *glutamicum* *dcas9* expression vector, pEC-XK-p15A expressing dcas9(K848A/K1003A/ R1060A), p15A ori | This study | | | |
| pXMJPsod-X-sgRNA | | *C*. *glutamicum* sgRNA expression vector | This study | | | |
| Primers | | | | | | |
| pycF | CGCGAGCTCaaggagTACTCTAGTGTCGACTCACACAT  (*Sac*I) | | | PCR for *C. glutamicum pyc* |  |  |
| pycR | CGCTCTAGATTAGGAAACGACGACGAT(*Xba*I) | | |  |  |  |
| pyc458F | ATTGCCGATCACTCGCACCTCCTTCAGG | | | Site-directed mutagenesis  for *pyc* |  |  |
| pyc458R | GAAGGAGGTGCGAGTGATCGGCAATGAAT | | |  |  |  |
| Kgd-TTG-UPF | CGGGATCCTCGACGAAGCCACCTCCACCC （BamHI） | | | Replacing the native kgd start codon with TTG |  |  |
| Kgd-TTG-UPR | CGCTGCTCAAGGCAGGTACTCGCCT | | |  |  |  |
| Kgd-TTG-DNF | GTACCTGCCTTGAGCAGCGCTAGTA | | |  |  |  |
| Kgd-TTG-DNR | GCTCTAGAGCAGATATCCATGTTCTTCGCG （XbaI） | | |  |  |  |
| P15AF | *CCACTGAGCGTCAGACCCCGTAGAAACCT*CCTGTTCAGCTACTGAC | | | PCR for p15A *ori* |  |  |
| P15AR | *CAAAAGGCCAGGAACCGTAAAAAGGCG*GGCTCGCCACTTCGGGCTC | | |  |  |  |
| PEC-AF | *GCTCATGAGCCCGAAGTGGCGAGCCC*GCCTTTTTACGGTTCCTGGC | | | PCR for pEC-XK99E |  |  |
| PEC-AR | *CACCCCGTCAGTAGCTGAACAGGAGG*TTTCTACGGGGTCTGACGCT | | |  |  |  |
| dCas9*F | TCCCCCGGGgaaaggaCGCTTATGGATAAGAAATAC (*Xma*I) | | | PCR for dCas9* |  |  |
| dCas9*R | GCTCTAGAGCTCAGTCACCTCCTAGCTGACTCAAA (*Xba*I) | | |  |  |  |
| psodG-F | AAAGATCTGCTCTAGAGCGTCGAC | | | PCR for pXMJPsod | |  |
| psodG-R | CTCCCAATGATAGGCCTTTTTGTTG | | |  | |  |
| sgRNAF | *GGCCTATCATTGGGAG*GTTTTAGAGCTAGAAATAGCAAG | | | PCR for sgRNA |  |  |
| sgRNAR | *CTAGAGCAGATCTTT*AAAAAAAGCACCGACTCG | | |  |  |  |
| carBN20F | ACATGCCTGGCCAATGACGAGTTTTAGAGCTAGAAATAGC | | | For *carB* sgRNA |  |  |
| thrB1N20F | AGAAGATCCAGGTACCGTGAGTTTTAGAGCTAGAAATAGC | | | For *thrB1* sgRNA |  |  |
| coaAN20F | GCTGAAATCTGGTGTGCGTGGTTTTAGAGCTAGAAATAGC | | | For *coaA* sgRNA |  |  |
| glnAN20F | TGAACTTGACAATTTCTTCCGTTTTAGAGCTAGAAATAGC | | | For *glnA* sgRNA |  |  |
| nadDN20F | CCATGATGCCAATGCGAGCGGTTTTAGAGCTAGAAATAGC | | | For *nadD* sgRNA |  |  |
| hemHN20F | TGCCGACAGGTGCTTGTACTGTTTTAGAGCTAGAAATAGC | | | NCgl2508 |  |  |
| xylBN20F | CAAGCAAAGCCTTGCAGGATGTTTTAGAGCTAGAAATAGC | | | For *xylB* sgRNA |  |  |
| guaAN20F | CGAAGTCAACGACGAGTACTGTTTTAGAGCTAGAAATAGC | | | For *guaA* sgRNA |  |  |
| accBCN20F | CGCGGATTGCAATCTCACCAGTTTTAGAGCTAGAAATAGC | | | For *accBC* sgRNA |  |  |
| accDCN20F | TTCCAAGAAATGAAGCTGTCGTTTTAGAGCTAGAAATAGC | | | For *accDC* sgRNA |  |  |
| purLN20F | CGCAATCAAGACCCCTGAGCGTTTTAGAGCTAGAAATAGC | | | For *purL* sgRNA |  |  |
| purQN20F | ATCGGTGTCATTACCTTCCCGTTTTAGAGCTAGAAATAGC | | | For *purQ* sgRNA |  |  |
| pacC1N20F | GTCGAGCAGATCCGCATGTTGTTTTAGAGCTAGAAATAGC | | | For *panC1* sgRNA |  |  |
| panC2N20F | GTCGAGCAGATCCGCATGTTGTTTTAGAGCTAGAAATAGC | | | For *panC* sgRNA |  |  |
| pknGN20F | CCCTTTCGACGATGACGATGGTTTTAGAGCTAGAAATAGC | | | For *pknG* sgRNA |  |  |
| pobAN20F | CGATTGATTCCACACCTTGAGTTTTAGAGCTAGAAATAGC | | | For *pobA* sgRNA |  |  |
| aldHN20F | TTCAGACTCGCATTGGTGCGGTTTTAGAGCTAGAAATAGC | | | NCgl0437 |  |  |
| fabG1N20F | ACCAAGTCCGCGTGCACCGCGTTTTAGAGCTAGAAATAGC | | | For *fabG1* sgRNA |  |  |
| adhCN20F | GCTGCTACTTGCCGTGCCATGTTTTAGAGCTAGAAATAGC | | | For *adh* sgRNA |  |  |
| gorN20F | ATCAAACTCTGGTCCAGGAAGTTTTAGAGCTAGAAATAGC | | | For *gor* sgRNA |  |  |
| dxrN20F | GCCTGCGAAATAACGAGGTCGTTTTAGAGCTAGAAATAGC | | | For *dxr* sgRNA |  |  |
| asdN20F | GAATTCAATCTTACGGCCTGGTTTTAGAGCTAGAAATAGC | | | For *asd* sgRNA |  |  |
| proAN20F | CAGGATTGCGTTCTTCGGTCGTTTTAGAGCTAGAAATAGC | | | For *proA* sgRNA |  |  |
| NCgl2558N20F | GCGCATCTCCATTGGGTCACGTTTTAGAGCTAGAAATAGC | | | For *NCgl2588* sgRNA |  |  |
| thyXN20F | GTTGACCACTCAACATCAGCGTTTTAGAGCTAGAAATAGC | | | For *thyX* sgRNA |  |  |
| aroEN20F | AGTATTCTCCTCGGCCTAATGTTTTAGAGCTAGAAATAGC | | | For *aroE* sgRNA |  |  |
| sirN20F | CAACAACCACCGGAAGTGCCGTTTTAGAGCTAGAAATAGC | | | For *sir* sgRNA |  |  |
| NCgl0503N20F | TTCGGCACCGTTCATCTTGAGTTTTAGAGCTAGAAATAGC | | | For *NCgl0503* sgRNA |  |  |
| ddhN20F | CAACATCCGCGTAGCTATCGGTTTTAGAGCTAGAAATAGC | | | For *ddh* sgRNA |  |  |
| ilvCN20F | CGCTGACCTCTCCTTGATCCGTTTTAGAGCTAGAAATAGC | | | For *ilvC* sgRNA |  |  |
| qorN20F | CACCGGCGGACCAGAGGTGTGTTTTAGAGCTAGAAATAGC | | | For *qor* sgRNA |  |  |
| trxBN20F | ATTCATGATGTCGCCATCATGTTTTAGAGCTAGAAATAGC | | | For *trxB* sgRNA |  |  |
| NCgl0200N20F | GCAAGCCATCGTCCAAACTGGTTTTAGAGCTAGAAATAGC | | | For *NCgl0200* sgRNA |  |  |

References

Cress, B.F., Toparlak, O.D., Guleria, S., Lebovich, M., Stieglitz, J.T., Englaender, J.A., Jones, J.A., Linhardt, R.J., and Koffas, M.a.G. (2015). CRISPathBrick: Modular combinatorial assembly of type II-A CRISPR arrays for dCas9-mediated multiplex transcriptional repression in *E. coli*. *ACS Synthetic Biology* 4**,** 987-1000.

Guzman, L.M., Belin, D., Carson, M.J., and Beckwith, J. (1995). Tight regulation, modulation, and high-level expression by vectors containing the arabinose PBAD promoter. *Journal of Bacteriology* 177**,** 4121-4130.

Kirchner, O., and Tauch, A. (2003). Tools for genetic engineering in the amino acid-producing bacterium *Corynebacterium glutamicum*. *Journal of Biotechnology* 104**,** 287-299.

Jiang, Y., Chen, B., Duan, C., Sun, B., Yang, J., and Yang, S. (2015). Multigene editing in the *Escherichia coli* genome via the CRISPR-Cas9 system. *Applied and Environmental Microbiology* 81**,** 2506-2514.

Jiang, L.Y., Chen, S.G., Zhang, Y.Y., and Liu, J.Z. (2013a). Metabolic evolution of *Corynebacterium glutamicum* for increased production of L-ornithine. *BMC Biotechnology* 13**,** 47.

Jiang, L.Y., Zhang, Y.Y., Li, Z., and Liu, J.Z. (2013b). Metabolic engineering of *Corynebacterium glutamicum* for increasing the production of L-ornithine by increasing NADPH availability. *Journal of Industrial Microbiology & Biotechnology* 40**,** 1143-1151.

Yim, S.S., An, S.J., Kang, M., Lee, J., and Jeong, K.J. (2013). Isolation of fully synthetic promoters for high-level gene expression in *Corynebacterium glutamicum*. *Biotechnology Bioengineering* 110**,** 2959-2969.
